# Supplementary material for: Calcium/Calmodulin Dependent Protein Kinase Kinase 2 Regulates the Expansion of Tumor-Induced Myeloid-Derived Suppressor Cells
Source: Front Immunol. 2021 Oct 12;12:754083. doi: 10.3389/fimmu.2021.754083 (PMC8546266; doi:10.3389/fimmu.2021.754083)
Supplement: Supplementary file 1 [file DataSheet_1.pdf]

Supplemental material

Calcium/Calmodulin Dependent Protein Kinase Kinase 2 Regulates the Expansion of Tumor-induced Myeloid-Derived Suppressor Cells

Wei Huang, Yaping Liu, Anthony Luz, Mark Berrong, Joel N Meyer, Yujing Zou, Excel Swann, Pasupathi Sundaramoorthy, Yubin Kang, Shekeab Jauhari, William Lent, Nelson Chao and Luigi Racioppi.

Supplementary Tables

Table 1: Antibodies and reagents used for flow cytometry.

Antibody/Reagent	Clone	Fluochorme	Company
anti-mouse CD11c	N418	FITC	BioLegend
anti-mouse/human CD11b	M1/70	PE	BioLegend
anti-mouse Ly6G	1A8	PE-Cy7	BioLegend
anti-mouse Ly6C	HK1.4	PerCP-Cy5.5	Life Technologies
anti-mouse F4/80	BM8	APC	BioLegend
anti-mouse I-A/I-E	M5/114/15/2	APC-Cy7	Life Technologies
anti-mouse CD69	H1.2F3	FITC	BioLegend
anti-mouse CD8a	53-6.7	PE-Cy7	BioLegend
anti-mouse CD4	GK1.5	APC-Cy7	BioLegend
anti-mouse CD25	PC51	APC	BD Biosciences
Fixable Viability Dye eFluor® 450			Life Technologies
Cell Apoptosis Detection Kit			BD Biosciences
CellTrace Violet Cell Proliferation Kit			Life Technologies
H2DCFDA			Life Technologies
MitoTracker Green			Life Technologies

*Antibodies and reagents were used according to the manufacturer's instruction.

Table 2: Antibodies used for Western Blot

Antibody/Reagent	Company
purified mouse anti-CaM kinase kinase	BD Biosciences
anti-phosphor-AMPK alpha (T172 40H9) rabbit mAb	Cell Signaling Technology
anti-AMPK alpha (F6) mouse mAb	Cell Signaling Technology
anti-phopho-Stat3 (Tyr705 D3A7) rabbit mAb	Cell Signaling Technology
anti-Stat3 (124H6) mouse mAb	Cell Signaling Technology
anti-β actin mouse mAb	Cell Signaling Technology
anti-mouse IgG Alexa Fluor 680	Invitrogen
anti-rabbit IgG IRDye800 conjugated	Rockland Immunochemicals

Table 3: Primers used for real-time PCR

Primer	Sequence (5'-3')
mGAPDH-F	CCT GGA GAA ACC TGC CAA GTA TG
mGAPDG-R	AGA GTG GGA GTT GCT GTT GAA GTC
mARG1-F	GCA AGG TGA TGG AAG AGA C
mARG1-R	CAT CGA CAT CAA AGC TCA GG
mNOS2-F	GCA AAC ATC ACA TTC AGA TCC C
mNOS2-R	TCA GCC TCA TGG TAA ACA CG
mCAMKK2-F	CAT GAA TGG ACG CTG C
mCAMKK2-R	TGA CAA CGC CAT AGG AGC C
mNrf2-F	CAG CAT AGA GCA GGA CAT GGA G
mNrf2-R	GAA CAG CGG TAG TAT CAG CCA G
mCAT-F	CGG CAC ATG AAT GGC TAT GGA TC
mCAT-R	AAG CCT TCC TGC CTC TCC AAC A
mRetnla-F	CAA GGA ACT TCT TGC CAA TCC AG
mRetnla-R	CCA AGA TCC ACA GGC AAA GCC A
mChi3l3-F	TAC TCA CTT CCA CAG GAG CAG G
mChi3l3-R	CTC CAG TGT AGC CAT CCT TAG G

Supplementary Figures

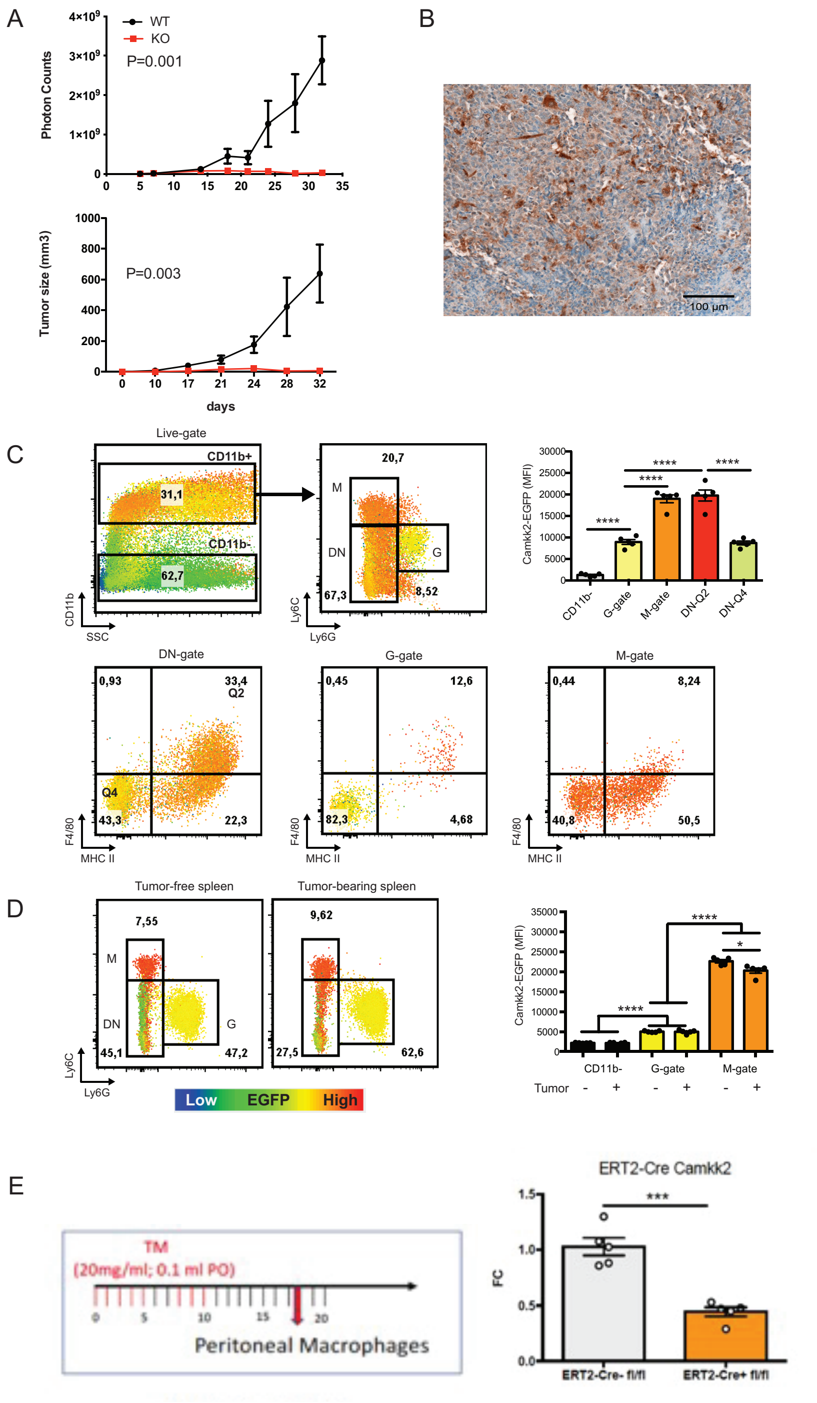

Figure S1. *Camkk2* promoter is active in myeloid cells associated with EG7-OVA tumors. (A) EG7-OVA cells (1 x 10^5 cells/mouse) were inoculated into the flank of WT and *Camkk2*$^{-/-}$ mice. Photon counts (upper panel) and tumor size (lower panel) were measured every 7 days and 3 days, respectively. (B-D) EG7-OVA cells (5 x 10^5

cells/mouse) were inoculated into the flank of (Tg)-*Camkk2*-EGFP reporter mice. Tumors were removed for histology and digested with collagenase/DNAaseI for FACS staining. (B) Histology of E.G7 tumor. Tumor was removed and stained for anti-EGFP antibody. Histology section was taken by microscope with 20x magnification. (C) FACS profiles and gating strategy of tumor-associated myeloid cells. Heatmaps refer to EGFP expression. Histogram quantified the mean fluorescence intensity (MFI) of EGFP in different myeloid subsets. (D) Spleens were removed from control EG7 bearing mice (normal and tumor, respectively) and stained for flow cytometry. Splenocytes were primarily gated on CD11b+ myeloid cells, and then sub gated to assess EGFP expression in myeloid subsets. Bars graphs reported mean and SEM of MFI (N=5 mice/group). * and **** refers to p<0.05 and 0.001, respectively. (E) *Camkk2* expression in peritoneal lavage macrophage derived from tamoxifen-treated ERT2-Cre $Camkk2^{fl/fl}$ mice. ERT2-Cre- $Camkk2^{fl/fl}$ and ERT2-Cre+ $Camkk2^{fl/fl}$ were treated with 8 doses of tamoxifen (TM) PO, according to scheme shown in the upper panel. After 8 days from the last TAM dose, mice were euthanized, and peritoneal lavage macrophages were isolated. *Camkk2* RNA was assessed by qPCR (left and right panels, respectively). Average and SEM are shown (n = 5 mice/genotype). *** refer to p-value < 0.005. T-test was used to calculate p-values.

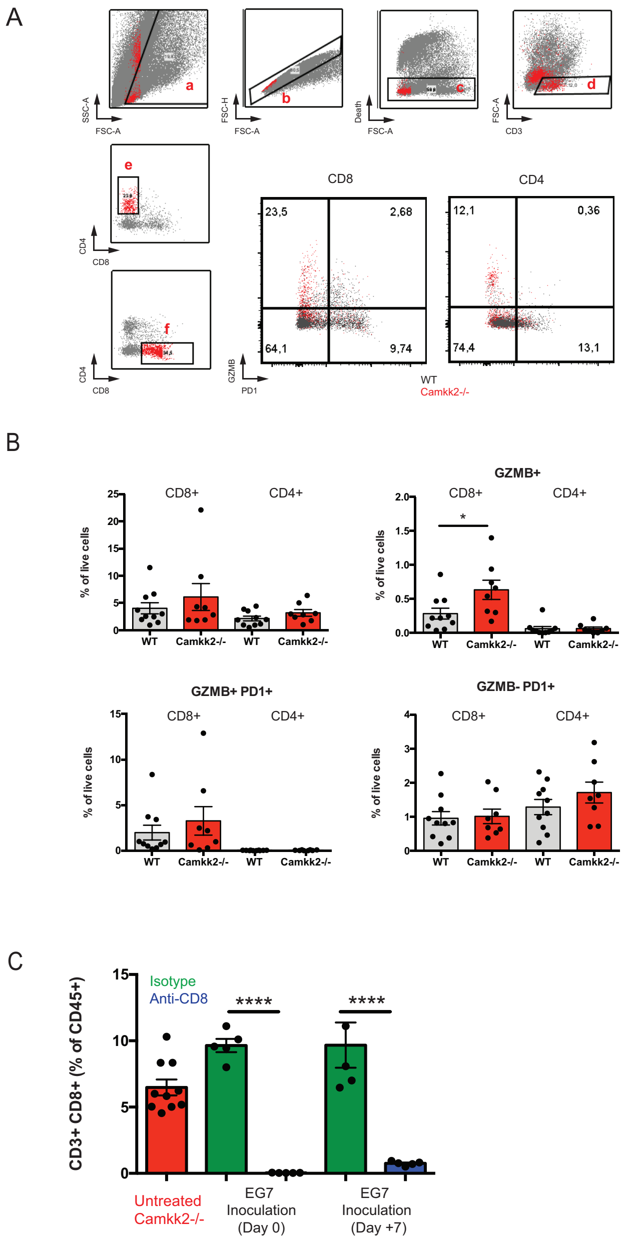

Figure S2. T-cell staining of E.G7 tumor from WT and *Camkk2*$^{-/-}$ mice. (A) Gating strategy and representative flow cytometry plots of T cells from the E.G7-OVA tumors collected from WT and *Camkk2*$^{-/-}$ mice. (B) Percentages of Tumor-infiltrating T cell subsets in tumors from WT and *Camkk2*$^{-/-}$ mice. * refers to $p<0.05$; n=7-10. Combined from two experiments. (C) Percentage of CD8^{+} T cells in the blood of anti-CD8 treated and untreated *Camkk2*$^{-/-}$ mice. ****Refer to $p<0.001$.

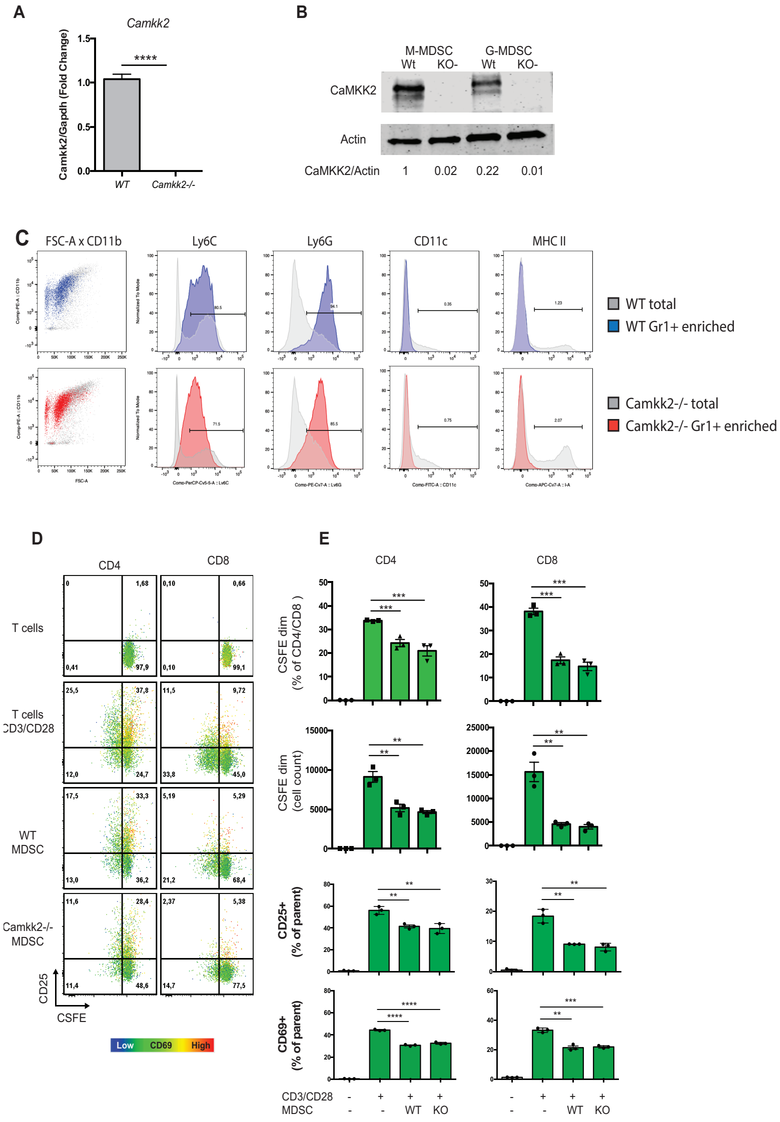

Figure S3. CaMKK2 deletion does not impair MDSCs ability to suppress T cell proliferation. (A-B) CaMKK2 is expressed in bone marrow derived MDSCs. (A) Expression of *Camkk2* mRNA in unsorted MDSC. (B) Immunoblots from FACS sorted M-MDSC and G-MDSC. (C-E) Gr1-enriched MDSC were co-cultured with CFSE-labeled syngeneic T-cells in the presence of anti-mouse CD3/CD28-coated beads. At day 3, cells were recovered, stained, and analyzed by FACS. (C) Immune profiles of WT and Camkk2-/- MDSC stained before and after enrichment with anti-Gr1 antibody. (D) Representative dot plot profiles of CD3+ gated cells. Heatmap represented CD69 expression. (E, top panels) Percentage and absolute numbers of CFSE-dim proliferated T-cells from different culture conditions. (E low panels) Percentage of CSFE+, $CD25^+$ and $CD69^+$ cells in $CD3^+CD4^+$ and $CD3^+CD8^+$ (left and right, respectively). N=3. **, *** and *** refers to p<0.01, 0.001 and 0.0001, respectively.

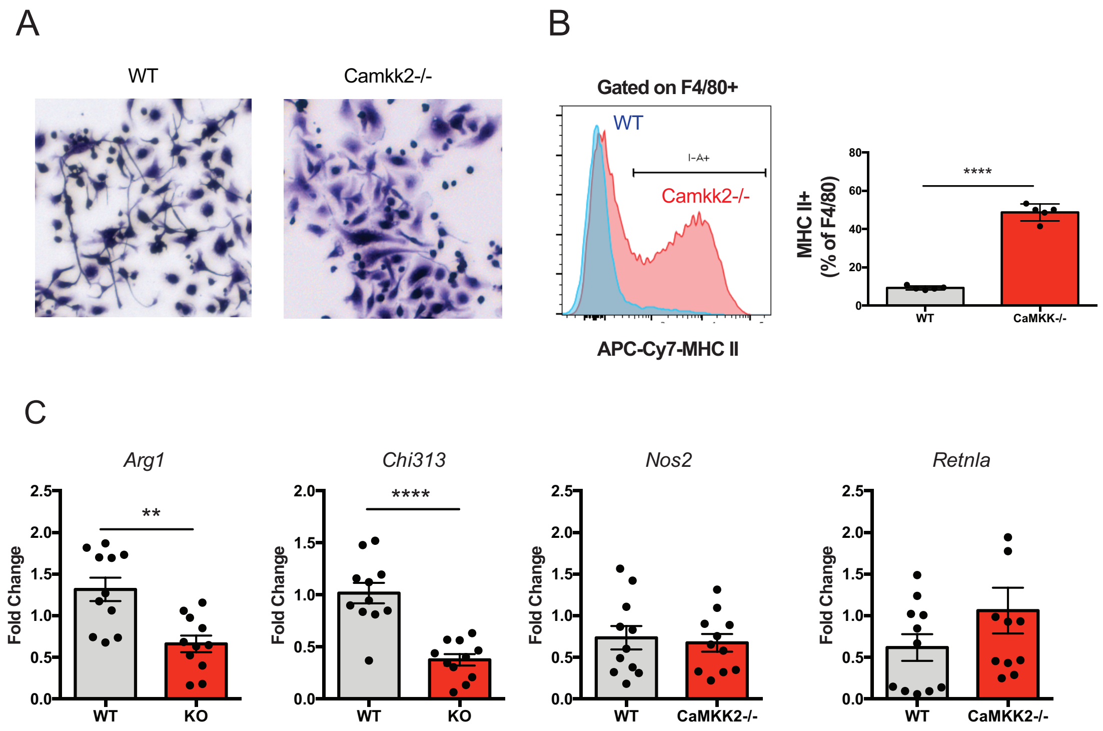

Figure S4. CaMKK2 regulates terminal differentiation and polarization of bone marrow cells macrophages. Bone marrow cells from WT and *Camkk2*$^{-/-}$ mice were cultured in the presence of GM-CSF and E.G7-OVA TCM. After 4 days, non adherent MDSC were collected and analyzed by flow cytometry. Adherent cells were further analyzed for phenotype and gene expression. (A) Crystal violet staining. (B) FACS profiles and quantitation of major histocompatibility class II I-A molecules (MHC II) on CD11b^{+}Ly6C^{-}Ly6G^{-}F4/80^{+} gated cells. N=5. Repeated three times. (E) Quantitation of *Arg-1*, *Chi313*, *Nos2*, and *Retnla* by qPCR n=11; combined from three independent experiments. * $p<0.05$. ** $p<0.01$. *** $p<0.001$, **** $p<0.0001$.

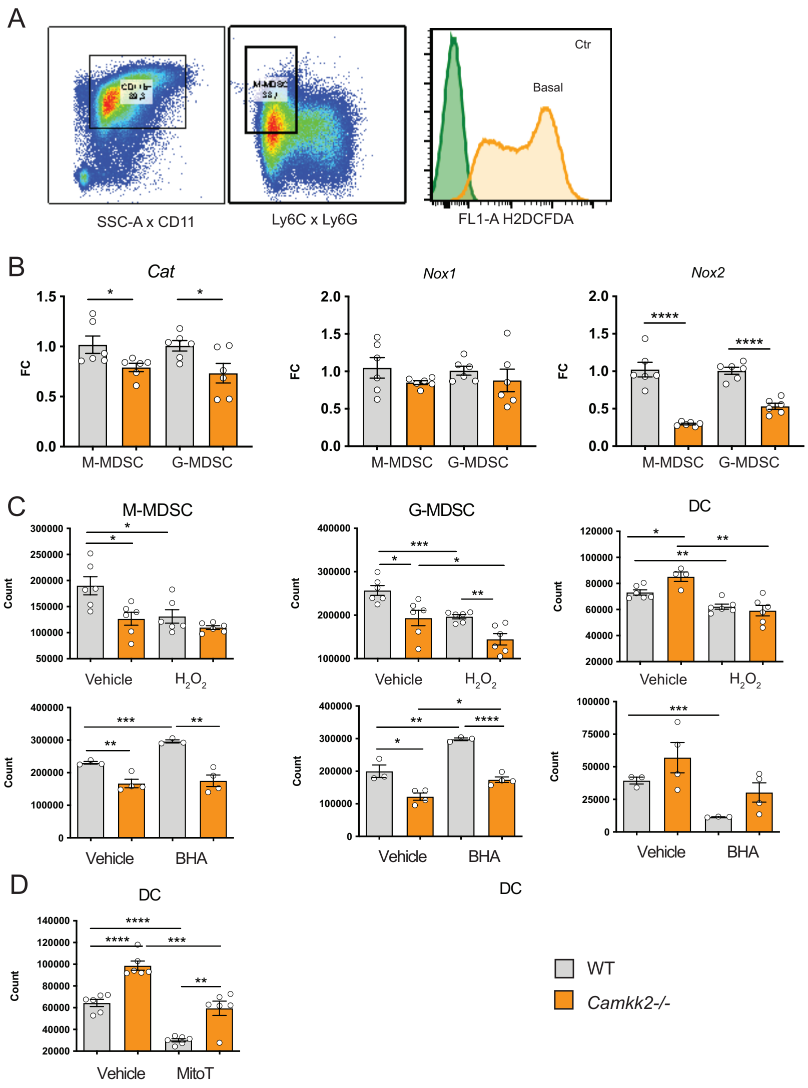

Figure S5. Radical oxygen species (ROS) regulates generation and fate of bone marrow generated MDSCs. MDSCs were generated from bone marrow nucleated cells of WT and *Camkk2*$^{-/-}$ mice femurs. (A) Gating strategy and representative FACS profiles of cells stained for MDSC surface markers and loaded with H2DCFDA to detect intracellular ROS. (B) M-MDSC and G-MDSC were purified by sorting and gene expression was determined by qPCR (*Cat*: catalase; *Nox1* and *Nox2*: NADPH Oxidase 1 and 2, respectively). Fold changes (FC) were normalized to WT average percentage. (C) Yield of MDSC generated from bone marrow cells in the presence or absence of 1μM H_2O_2 (upper) or 10μM BHA (lower). Drugs were added on Day0 and Day3 into culture media. (D) Yield of DC (DN, CD11c^{+} MHC II^{+}) in MDSC cultures generated in the presence or absence of 20μM Mito Tempo added on Day 0 and Day 3**.** *, **, ***, and **** refer to $p<0.05$, $p< 0.01$, $p<0.001$, $p<0.0001$, respectively.
